# Genetic association analysis of lipid-lowering drug target genes in chronic kidney disease

**DOI:** 10.3389/fendo.2024.1434145

**Published:** 2025-01-14

**Authors:** Yi Zhang, Guangyang Ou, Lei Peng, Jian Pan, Shaohua Zhang, Jianguo Shi

**Affiliations:** ^1^ Department of Urology, The First Affiliated Hospital of Jinzhou Medical University, Jinzhou Medical University, Jinzhou, Liaoning, China; ^2^ Department of Urology, The Third Affiliated Hospital of Jinzhou Medical University, Jinzhou Medical University, Jinzhou, Liaoning, China; ^3^ Department of Cardiology, Hunan University of Chinese Medicine, Changsha, China; ^4^ Motor Robotics Institute (MRI), South China Hospital, Health Science Center, Shenzhen University, Shenzhen, China

**Keywords:** chronic kidney disease, lipid-lowering drug, lipids, Mendelian randomization analysis, estimated glomerular filtration rate

## Abstract

**Objective:**

The impact of lipid-lowering medications on chronic kidney disease (CKD) remains a subject of debate. This Mendelian randomization (MR) study aims to elucidate the potential effects of lipid-lowering drug targets on CKD development.

**Methods:**

We extracted 11 genetic variants encoding targets of lipid-lowering drugs from published genome-wide association study (GWAS) summary statistics, encompassing LDLR, HMGCR, PCSK9, NPC1L1, APOB, ABCG5/ABCG8, LPL, APOC3, ANGPTL3, and PPARA. A Mendelian randomization analysis was conducted targeting these drug-related genes. CKD risk was designated as the primary outcome, while estimated glomerular filtration rate (eGFR) and blood urea nitrogen (BUN) were assessed as secondary outcomes. Additionally, mediation analysis was performed utilizing 731 immune cell phenotypes to identify potential mediators.

**Results:**

The meta-analysis revealed a significant association between ANGPTL3 inhibitors and a reduced risk of CKD (OR [95% CI] = 0.85 [0.75-0.96]). Conversely, LDLR agonists were significantly linked to an increased risk of CKD (OR [95% CI] = 1.11 [1.02-1.22]). Regarding secondary outcomes, lipid-lowering drugs did not significantly affect eGFR and BUN levels. Mediation analysis indicated that the reduction in CKD risk by ANGPTL3 inhibitors was mediated through modulation of the immune cell phenotype, specifically HLA-DR on CD14+ CD16+ monocytes (Mediated proportion: 4.69%; Mediated effect: -0.00899).

**Conclusion:**

Through drug-targeted MR analysis, we identified a causal relationship between lipid-lowering drug targets and CKD. ANGPTL3 and LDLR may represent promising candidate drug targets for CKD treatment.

## 1 Introduction

Chronic kidney disease (CKD) is characterized by a glomerular filtration rate (GFR) falling below 60 mL/min per 1.73 m² without clear cause, or the presence of markers of kidney damage, such as proteinuria, for a duration of three months or more (1). Epidemiological data reveal that CKD affects over 10% of the global population, with approximately 9.6% of non-hospitalized adults in the United States afflicted by this condition (2–4). Similar prevalence rates have been observed in Europe, Australia, and Asia (5–7). This widespread incidence of CKD imposes substantial economic burdens, with treatment and care costs surpassing those of many other prevalent diseases (8). CKD is intricately linked with cardiovascular risk factors, including hyperlipidemia, diabetes, and hypertension. Research has established a frequent co-occurrence of CKD and cardiovascular diseases, often referred to as cardiorenal syndrome. Both conditions are underpinned by shared pathogenic mechanisms, such as endothelial dysfunction, oxidative stress, and systemic inflammation of the capillary walls in CKD patients (9, 10). Thus, CKD and cardiovascular diseases are interconnected through common risk factors. Lipids play a crucial role in this association.

Treatments for hyperlipidemia commonly include nicotinic acid, statins, fibrates, and newer lipid-lowering drugs (11–14). Extensive research has been conducted on statins and their relationship with CKD or kidney function, yet these studies remain controversial. Some trials have confirmed renal protective effects and reduced proteinuria (15, 16), while other RCTs show no effect (17). However, the latest meta-analysis of statins and CKD included 33 RCTs, finding no significant differences in estimated glomerular filtration rate (eGFR) and serum creatinine levels between the statin and control groups (18). Additionally, lipid-lowering drugs are not limited to statins; non-statin lipid-lowering drugs lack large-scale randomized controlled trials with CKD, and there is still a need for systematic research on whether lipid-lowering drugs directly affect the progression of CKD and levels of eGFR and blood urea nitrogen (BUN).

With the advent of genome-wide association studies (GWAS), Mendelian randomization (MR) has emerged as a potent alternative to randomized controlled trials (RCTs) for elucidating causal relationships. The random allocation of genetic variations (alleles) during meiosis ensures that participants in MR studies are effectively “randomized” based on their alleles. This process mirrors the random assignment in RCTs, where individuals are allocated to either the treatment or control group (19, 20). Such inherent randomization in MR studies significantly reduces the influence of confounding factors, offering a robust approach compared to other research methodologies. Drug-targeted MR analysis has gained traction as a method for inferring the effects of drugs targeting protein-coding genes—such as antagonists, agonists, or inhibitors—on disease risk (21). This approach significantly enhances the evaluation of drug therapy potentials and facilitates the development of novel pharmaceuticals.

We employed drug-targeted MR analysis to investigate the impact of lipid-lowering drugs on CKD and to explore the potential effects of lipid-lowering drug targets on eGFR and BUN.

## 2 Materials and methods

### 2.1 Study design

**Figure 1:**
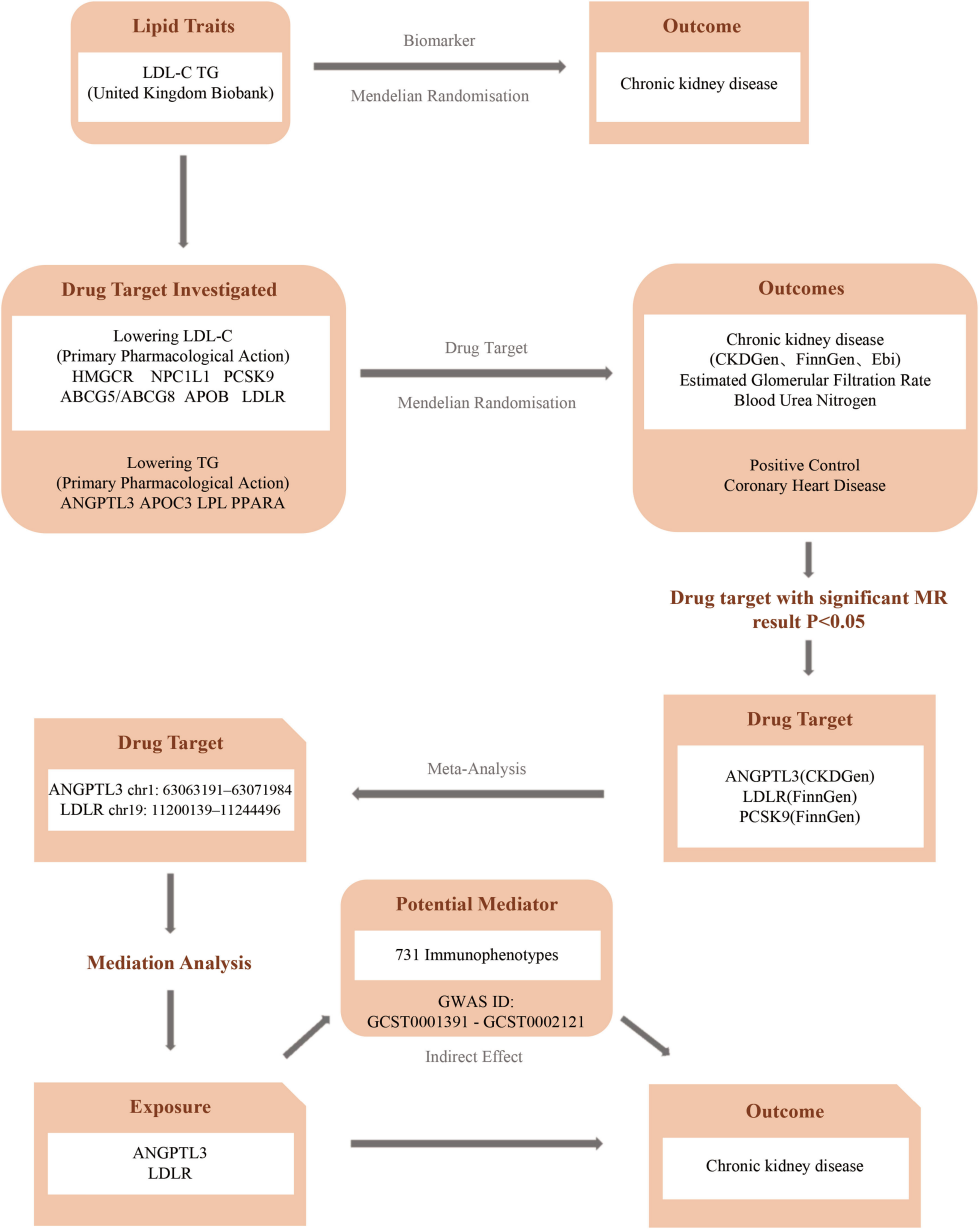
Flowchart of the study. (CKDGen, Chronic Kidney Disease Genetics Consortium; FinnGen, FinnGen Study; EBI, European Bioinformatics Institute).

This investigation employs a two-sample Mendelian randomization approach to elucidate the genetic interplay between lipid-lowering drug target genes and CKD. MR relies on three key assumptions: 1) the genetic instrument is strongly associated with the exposure, 2) it is independent of confounders, and 3) it affects the outcome only through the exposure, not via other pathways (22, 23). A schematic overview of the study design is presented in **Figure 1**.

### 2.2 Genetic variant selection

Adhering to the latest dyslipidemia treatment guidelines, we selected a range of widely used lipid-lowering drugs and the most recent therapeutic methods. These included statins, ezetimibe, PCSK9 inhibitors, bile acid sequestrants, mipomersen, fibrates, ANGPTL3 inhibitors, and antisense oligonucleotides targeting apolipoprotein C-III (APOC3) mRNA (24, 25). Utilizing the DrugBank database, we identified the genes encoding the pharmacological targets of these drugs. These target genes were classified into two groups: those that reduce LDL cholesterol (i.e., LDLR, HMGCR, NPC1L1, PCSK9, APOB, ABCG5, and ABCG8) and those that lower triglycerides (i.e., LPL, APOC3, ANGPTL3, and PPARA), as detailed in **Table 1**.

**Table 1:** Characteristics of Lipid-lowering drug target genes.

Primary pharmacological action	Drug class	Related Drugs	Drug targets	Target genes	Gene region (GRCh3.p13 by NCBI Gene)	SNPs
Reduced LDL-C	Key regulator	RGX-501^a^	LDL Receptor^b^	LDLR	chr19: 11200139–11244496	45
	HMG-CoA reductase inhibitors	Lovastatin Simvastatin Atorvastatin Rosuvastatin Pravastatin Fluvastatin	HMG-CoA reductase	HMGCR	chr5: 74632993–74657941	19
	Proprotein convertase subtilisin/kexin type 9 inhibitors	Alirocumab Evolocumab	Proprotein convertase subtilisin/kexin type 9	PCSK9	chr1: 55505221–55530525	33
	Cholesterol absorption inhibitors	Ezetimibe	Niemann-Pick C1-like protein 1	NPC1L1	chr7: 44552134–44580929	6
	Antisense oligonucleotide targeting ApoB-100 mRNA	Mipomersen	Apolipoprotein B-100	APOB	chr2: 21224301–21266945	31
	Bile acid sequestrants	Colesevelam Colestipol Cholestyramine	ATP Binding Cassette Subfamily G Member 5/ATP Binding Cassette Subfamily G Member 8	ABCG5/ ABCG8^c^	chr2: 44039611–44065978 /44066110–44110127	20/19
Reduced TG	Key regulator		Lipoprotein Lipasea^b^	LPL	chr8: 19796764–19824770	50
	Antisense oligonucleotide targeting ApoC-III mRNA	Volanesorsen	Apolipoprotein C-III	APOC3	chr11: 116700623–116703788	36
	Angiopoietin-like 3 Inhibitor	Evinacumab	Angiopoietin-related protein 3	ANGPTL3	chr1: 63063191–63071984	21
	peroxisome proliferator receptor alpha activators	Fenofibrate Gemfibrozil Bezafibrate Clofibrate	Peroxisome proliferator-activated receptor-a	PPARA	chr22:46546429–46639653	2

SNPs, single-nucleotide polymorphisms, chr chromosome, mRNA messenger ribonucleic acid, LDL-C low-density lipoprotein cholesterol, TG triglyceride;

^a^ : RGX-501 is not yet approved for marketing and is being studied for the treatment of homozygous familial hypercholesterolemia; ^b^ : LDL receptor and lipoprotein lipase are central players in LDL-C and TG metabolisms and are extensively involved in the lipid-lowering action; ^c^ : Drug targets of bile acid sequestrants were not specified in the Drug Bank. They were identified from a previous study.

Summary data for LDL-C and TG levels were derived from two extensive GWAS meta-analyses (26). Instrumental variables representing each lipid-lowering drug target were employed to model the effects of these interventions on lipid levels. Single nucleotide polymorphisms (SNPs) located within ±100 kb of the drug target loci and significantly associated with LDL-C or TG levels (P < 5×10^−8^) were selected as instrumental variables. To mitigate potential bias, SNPs were filtered based on an effect allele frequency (eaf) > 0.01. To minimize the impact of strong linkage disequilibrium, a linkage disequilibrium(LD) threshold (r2 < 0.3) was set. To avoid potential confounders, we examined each relevant SNP in the LDtrait Tool database to assess confounding factors associated with it (P <5e-8) (27, 28), such as age, hypertension, diabetes, proteinuria, and environmental risk factors like dietary salt intake and pollution (29), excluding SNPs highly correlated with confounding factors.

### 2.3 Outcome

We utilized coronary heart disease (CHD) and CKD as outcomes for our drug-targeted MR analysis. CHD served as a positive control dataset to validate the feasibility and effectiveness of the lipid-lowering drug targets. The CHD dataset was sourced from GWAS summary statistics, comprising 184,305 participants, including 60,801 cases and 123,504 controls (30). For CKD, the primary outcome, instrumental variables’ summary statistics were obtained from the latest Chronic Kidney Disease Genetics Consortium (CKDGen Consortium) database (31), incorporating relevant GWAS datasets from FinnGen and the ebi database (32). Secondary outcomes included eGFR and BUN, also from the CKDGen Consortium (31). Detailed information can be found in **Supplementary Table 1**.

To investigate the mediating role of immune cells, we accessed comprehensive GWAS data on immunity. Summary statistics for each immune trait were sourced from the publicly available GWAS Catalog, with accession numbers ranging from GCST9001391 to GCST0002121 (33). We incorporated a total of 731 immunophenotypes, spanning various categories: absolute cell counts (n = 118), median fluorescence intensities (MFI) representing surface antigen levels (n = 389), morphological parameters (MP) (n = 32), and relative cell counts (n = 192).

### 2.4 Estimation of causal effects

We estimated the causal effects between drug targets and CKD using the inverse variance-weighted method (IVW). Additionally, additional analyses were conducted using the weighted median and weighted model averaging (34–37). In all three analyses, statistically significant IVW results combined with consistent directions provided sufficient evidence of causal effects.

### 2.5 Meta-analysis

The odds ratio (OR) served as the primary combined statistic (38). Studies exhibiting significant heterogeneity, defined as I² > 50%, were analyzed using a random effects model. Conversely, studies with I² < 50% were considered homogeneous and analyzed using a fixed effects model. Data calculations were performed using the ‘meta’ package in R Studio, and the plots were generated using GraphPad Prism 9.

### 2.6 Quality controls

Heterogeneity was evaluated using both the MR Egger and Inverse Variance Weighted (IVW) methods. Cochran’s Q test assessed the heterogeneity of genetic instruments, with a p-value > 0.05 indicating no significant heterogeneity. The MR Egger regression was employed to evaluate horizontal pleiotropy, where a p-value > 0.05 suggested no evidence of pleiotropy (39, 40). To ensure result robustness, a leave-one-out analysis was conducted, sequentially removing each SNP to assess the stability of the IVW results.

### 2.7 Mediation MR analysis

Given the intricate relationship between the immune system and CKD progression, it is plausible that immune cells mediate the effects of lipid-lowering drugs on CKD. We employed a “two-sample” MR approach to evaluate the potential mediating effects of 731 immune cell phenotypes on CKD progression. This method, compared to multivariable MR approaches, reduces bias due to high linkage disequilibrium (LD) between genetic variants. All statistical analyses were conducted using RStudio software.

## 3 Results

### 3.1 Positive control analysis

**Figure 2:**
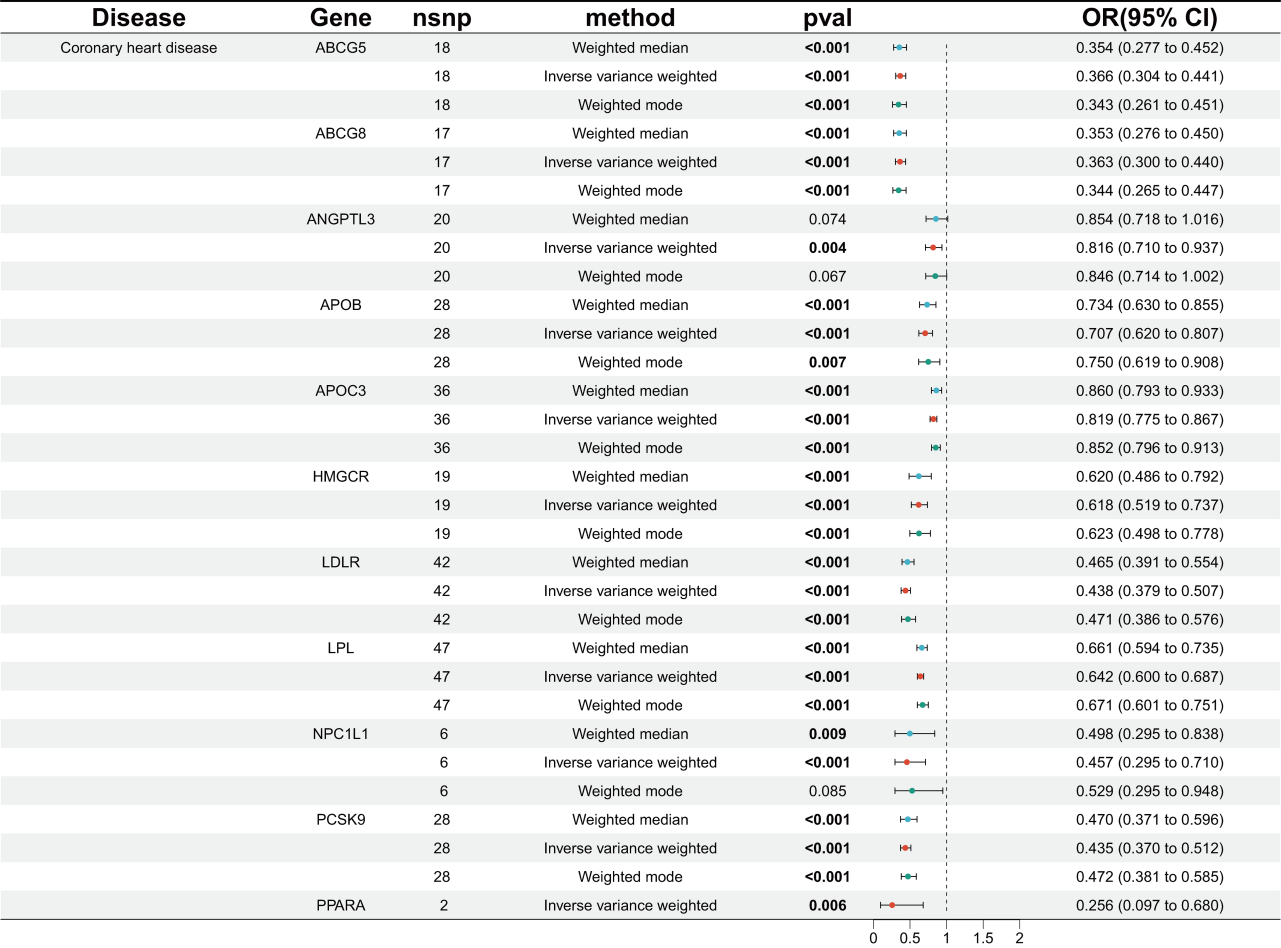
MR analysis of association between drug targets and CHD.

We identified genetic variants linked to various lipid-lowering drug targets, including LDLR agonists, HMGCR inhibitors, NPC1L1 inhibitors, PCSK9 inhibitors, APOB inhibitors, and ABCG5 agonists targeting LDL-C, as well as APOC3 inhibitors, LPL agonists, ANGPTL3 inhibitors, and PPARA agonists targeting TG. In the MR analysis evaluating CHD as the outcome, 11 drug-related target points significantly reduced the risk of CHD, as anticipated (**Figure 2**).

### 3.2 The causal relationship between drug targets and primary outcomes

According to the IVW results, ANGPTL3 inhibitors were significantly associated with a reduced risk of CKD in the CKDGen database (OR [95% CI] = 0.826 [0.714, 0.955], p = 0.010). Conversely, in the FinnGen database, LDLR agonists (OR [95% CI] = 1.394 [1.036, 1.875], p = 0.028) and PCSK9 inhibitors (OR [95% CI] = 1.404 [1.098, 1.795], p = 0.007) were significantly associated with an increased risk of CKD. The results of the MR analysis are summarized in **Table 2**.

**Table 2:** The effect of drug targets on CKD.

Drug target	Methods	CKD (CKDGen database)		CKD (FinnGen database )		CKD (Ebi database)	
		OR (95% CI)	P value	OR (95% CI)	P value	OR (95% CI)	P value
LDLR	Weighted median	1.129 (0.974 to 1.309)	0.107	**1.616 (1.131 to 2.311)**	**0.008**	1.206 (0.849 to 1.713)	0.296
	Inverse variance weighted	1.077 (0.973 to 1.192)	0.151	**1.394 (1.036 to 1.875)**	**0.028**	1.200 (0.882 to 1.633)	0.246
	Weighted mode	1.142 (0.988 to 1.319)	0.079	**1.743 (1.173 to 2.589)**	**0.009**	1.207 (0.838 to 1.740)	0.339
HMGCR	Weighted median	1.192 (0.937 to 1.514)	0.152	0.765 (0.434 to 1.348)	0.355	0.896 (0.612 to 1.310)	0.570
	Inverse variance weighted	1.151 (0.959 to 1.381)	0.132	0.816 (0.542 to 1.230)	0.332	0.844 (0.611 to 1.164)	0.301
	Weighted mode	1.196 (0.960 to 1.490)	0.134	0.755 (0.424 to 1.343)	0.352	0.962 (0.630 to 1.468)	0.861
PCSK9	Weighted median	1.028 (0.837 to 1.263)	0.793	1.284 (0.923 to 1.784)	0.137	1.243 (0.622 to 2.484)	0.538
	Inverse variance weighted	1.005 (0.870 to 1.160)	0.947	**1.404 (1.098 to 1.795)**	**0.007**	1.146 (0.596 to 2.206)	0.682
	Weighted mode	1.061 (0.871 to 1.292)	0.564	**1.352 (1.019 to 1.794)**	**0.046**	1.263 (0.503 to 3.172)	0.638
NPC1L1	Weighted median	0.949 (0.562 to 1.604)	0.846	1.017 (0.310 to 3.343)	0.977	0.494 (0.196 to 1.242)	0.134
	Inverse variance weighted	0.892 (0.524 to 1.518)	0.673	1.079 (0.415 to 2.803)	0.876	0.514 (0.243 to 1.090)	0.083
	Weighted mode	0.953 (0.533 to 1.704)	0.877	0.972 (0.276 to 3.430)	0.967	0.494 (0.183 to 1.337)	0.237
APOB	Weighted median	0.950 (0.829 to 1.088)	0.457	1.058 (0.762 to 1.470)	0.735	0.851 (0.660 to 1.098)	0.214
	Inverse variance weighted	0.943 (0.855 to 1.041)	0.246	1.039 (0.823 to 1.311)	0.750	0.829 (0.638 to 1.077)	0.160
	Weighted mode	0.930 (0.799 to 1.083)	0.358	1.083 (0.761 to 1.542)	0.661	0.887 (0.697 to 1.130)	0.350
ABCG5	Weighted median	0.899 (0.696 to 1.161)	0.413	0.538 (0.278 to 1.042)	0.066	0.944 (0.618 to 1.442)	0.791
	Inverse variance weighted	0.950 (0.788 to 1.145)	0.591	0.679 (0.397 to 1.161)	0.157	0.991 (0.682 to 1.439)	0.961
	Weighted mode	0.939 (0.722 to 1.223)	0.649	0.545 (0.288 to 1.031)	0.078	0.889 (0.580 to 1.362)	0.603
ABCG8	Weighted median	0.899 (0.709 to 1.141)	0.382	0.542 (0.288 to 1.018)	0.057	0.944 (0.614 to 1.453)	0.795
	Inverse variance weighted	0.967 (0.800 to 1.167)	0.725	0.696 (0.405 to 1.198)	0.191	0.991 (0.682 to 1.439)	0.961
	Weighted mode	0.937 (0.740 to 1.185)	0.593	0.538 (0.261 to 1.108)	0.111	0.889 (0.598 to 1.321)	0.576
LPL	Weighted median	0.998 (0.909 to 1.097)	0.974	1.254 (0.964 to 1.632)	0.092	0.926 (0.780 to 1.098)	0.377
	Inverse variance weighted	0.955 (0.894 to 1.020)	0.168	1.119(0.925 to 1.355)	0.248	0.939 (0.822 to 1.071)	0.348
	Weighted mode	0.993 (0.897 to 1.101)	0.901	1.242 (0.955 to 1.614)	0.115	0.940 (0.794 to 1.112)	0.479
APOC3	Weighted median	0.971 (0.896 to 1.051)	0.467	0.960 (0.778 to 1.185)	0.705	0.882 (0.743 to 1.046)	0.149
	Inverse variance weighted	0.993 (0.933 to 1.056)	0.818	0.947 (0.806 to 1.113)	0.511	0.897 (0.776 to 1.037)	0.142
	Weighted mode	0.978 (0.904 to 1.057)	0.575	0.961 (0.800 to 1.154)	0.671	0.880 (0.735 to 1.054)	0.213
ANGPTL3	Weighted median	0.856 (0.719 to 1.020)	0.081	1.136 (0.705 to 1.831)	0.600	0.845 (0.635 to 1.126	0.251
	Inverse variance weighted	**0.826 (0.714 to 0.955)**	**0.010**	0.977 (0.679 to 1.405)	0.899	0.850 (0.596 to 1.212)	0.369
	Weighted mode	0.842 (0.716 to 0.991)	0.054	1.032 (0.674 to 1.580)	0.887	0.847 (0.610 to 1.177)	0.396
PPARA	Wald ratio or Inverse variance weighted	2.061 (0.533 to 7.967)	0.294	0.221 (0.018 to 2.654)	0.234	0.818 (0.092 to 7.254)	0.857

Bold values indicate p < 0.05; LDLR, Low-Density Lipoprotein Receptor; HMGCR, 3-Hydroxy-3-Methylglutaryl-CoA Reductase; PCSK9, Proprotein Convertase Subtilisin/Kexin Type 9; NPC1L1, Niemann-Pick C1-Like 1; APOB, Apolipoprotein B; ABCG5/ABCG8, ATP Binding Cassette Subfamily G Member 5 / ATP Binding Cassette Subfamily G Member 8; LPL, Lipoprotein Lipase; APOC3, Apolipoprotein C3; ANGPTL3, Angiopoietin-Like 3; PPARA, Peroxisome Proliferator-Activated Receptor Alpha.

**Figure 3:**
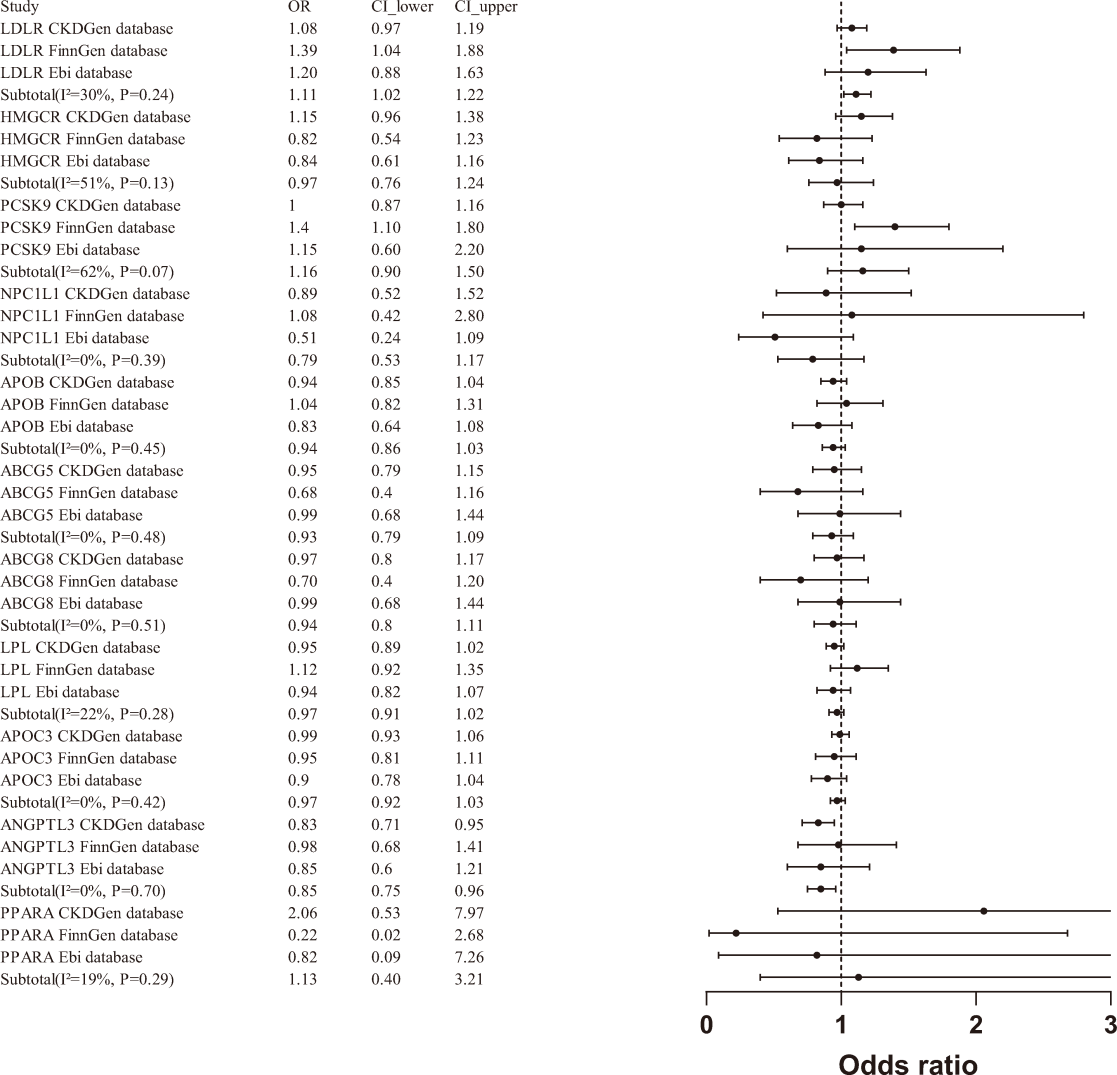
Meta analysis of association between drug targets and CKD.

A meta-analysis of the MR results from the three databases revealed a significant association between LDLR agonists and an increased risk of CKD (OR [95% CI] = 1.11 [1.02 - 1.22]), as well as a significant association between ANGPTL3 inhibitors and a reduced risk of CKD (OR [95% CI] = 0.85 [0.75-0.96]). However, when aggregating data from the three databases, PCSK9 inhibitors (OR [95% CI] = 1.16 [0.90-1.50]) and other drug targets were not significantly associated with the risk of CKD(**Figure 3**).

### 3.3 The causal relationship between drug targets and secondary outcomes

In the MR analysis with eGFR and BUN as outcomes, although multiple drug targets showed associations with these outcomes, their impact was negligible (**Supplementary Figures 9**, **10**).

### 3.4 Sensitivity analysis

Cochrane’s Q and MR Egger regression equations were employed to evaluate heterogeneity and horizontal pleiotropy levels. Significant horizontal pleiotropy was found when examining the causal relationship between LPL agonists and CKD (FinnGen) and eGFR (CKDGen) (CKD: p = 0.0311; eGFR: p = 0.0140) (**Supplementary Tables 13**, **14**). To ensure more reliable results, we applied stricter criteria to select instrumental variables, adjusting the linkage disequilibrium parameter from r2 < 0.3 to r2 < 0.2. Subsequent MR analysis did not reveal significant horizontal pleiotropy, and these updated results were included in the meta-analysis (**Supplementary Table 15**). Sensitivity analysis results for certain drug targets and outcomes exhibited heterogeneity (p < 0.05) (**Supplementary Tables 13**, **14**), while leave-one-out analysis indicated no significant differences in the results after the removal of any SNP (**Supplementary Figures 1**-**5**).

### 3.5 Mediation analysis

To investigate these intermediary pathways, we employed the coefficient product method in our mediation analysis, focusing on LDLR agonists and ANGPTL3 inhibitors. The findings suggest that ANGPTL3 inhibitors mitigate the risk of CKD by modulating the immune cell phenotype of HLA-DR on CD14+ CD16+ monocytes (Mediated proportion: 4.69%; Mediated effect: -0.00899) (**Supplementary Figure 11**, **Supplementary Table 16**), although the mediating effect remains relatively modest.

## 4 Discussion

Chronic kidney disease (CKD) is a prevalent condition that can lead to cardiovascular disease, renal failure, and other complications, imposing a significant societal burden due to its high prevalence and economic impact (5, 8). As a result, the epidemiology and pathogenesis of CKD are increasingly attracting global scholarly attention. Extensive experimental and clinical data indicate that hyperlipidemia is a critical shared risk factor for both CKD and cardiovascular diseases. Current research shows that abnormalities in lipid levels, akin to those in cardiovascular disease pathogenesis, are strongly associated with CKD through mechanisms such as endothelial dysfunction, inflammation, and the direct toxic effects of lipids on renal cells (9, 10, 41).

This investigation employed Mendelian randomization to elucidate the causal linkages between genetic targets of lipid-lowering therapeutics and the susceptibility to chronic kidney disease (CKD). Our findings reveal a notable correlation between the activation of the low-density lipoprotein receptor (LDLR) gene and an elevated risk of CKD, presenting an odds ratio (OR) of 1.11 (95% CI: 1.02 to 1.22). Conversely, inhibiting the ANGPTL3 gene correlates with a reduced CKD risk, indicated by an OR of 0.85 (95% CI: 0.75 to 0.96). These findings underscore the diverse impacts of lipid regulatory pathways on renal health, highlighting the complexity of lipid metabolism in CKD progression.

Emerging research supports a significant link between ANGPTL3 inhibition and reduced CKD risk, with several studies underscoring the protective role of ANGPTL3 inhibition in renal diseases (42–45). ANGPTL3 is a pivotal regulator of lipid metabolism, modulating the activity of key enzymes like lipoprotein lipase (LPL) and endothelial lipase, which influence plasma levels of triglycerides and high-density lipoprotein cholesterol. Recent studies have demonstrated that ANGPTL3 inhibitors not only improve lipid profiles but also offer renal benefits by mitigating lipid-induced glomerular injury and preserving endothelial function (46). Moreover, ANGPTL3’s role in lipid metabolism extends to regulating the production and clearance of VLDL, a vital carrier of triglycerides in the blood. By enhancing VLDL metabolism, ANGPTL3 inhibitors may improve the clearance of lipid particles, thereby reducing the risk of lipid accumulation and consequent renal damage (47).

Research has established that LDLR is a crucial receptor for removing LDL-C from plasma via endocytosis, and it is one of the genes associated with autosomal dominant familial hypercholesterolemia (48, 49). The activation of LDLR primarily facilitates the clearance of low-density lipoprotein cholesterol from the bloodstream, which is advantageous for reducing cardiovascular risk. However, our data indicate that LDLR activation may also exacerbate kidney disease. This adverse effect is likely due to the pathological accumulation of cholesterol in renal cells, leading to lipid toxicity—accumulated lipids disrupt cell function and induce cellular stress and damage (50–52). Further studies have shown that lipid accumulation in renal cells can promote cellular dysfunction and damage through mechanisms such as oxidative stress and inflammation, common pathways exacerbating kidney disease. For instance, excessive lipid accumulation in renal cells is linked with increased production of reactive oxygen species (ROS), which intensifies oxidative stress and accelerates the progression of diabetic nephropathy. This stress not only impacts cell vitality but also triggers inflammatory responses, further deteriorating renal function (52). Additionally, statin drugs often elevate circulating levels of lipoprotein (a) despite reducing LDL cholesterol levels, potentially heightening residual cardiovascular risks and advancing CKD progression (53). Understanding LDLR’s dual role in cardiovascular and renal health is crucial for developing targeted therapies that mitigate the adverse effects of cholesterol accumulation in the kidneys while leveraging its cardiovascular benefits.

In the FinnGen database, PCSK9 inhibitors (OR [95%] = 1.404 [1.098, 1.795], p=0.007) were significantly associated with an increased risk of CKD occurrence. However, after meta-analyzing data from three databases (CKDGen, FinnGen, Ebi), PCSK9 inhibitors and other drug targets were found to have no significant correlation with the risk of CKD occurrence (OR[95%]=0.1.16 [0.90-1.50]), reflecting the advantages of meta-analysis and the credibility of our results. The association between PCSK9 levels and the occurrence and progression of CKD remains contentious, and there is still no direct data on PCSK9 inhibitor administration in CKD patients. Observational studies on PCSK9 levels in CKD patients have shown conflicting results. Rogavec et al. and Elewa et al. found that PCSK9 levels were higher in subjects receiving statin therapy within the same CKD group (p < 0.05) (54, 55). However, Elewa et al. also observed a positive correlation between PCSK9 levels and total cholesterol, although it was not statistically significant (p = 0.078) (55). Abujrad et al. and Konarzweski et al. found no significant correlation between lipid parameters in CKD patients undergoing hemodialysis and PCSK9 levels (56, 57). Current studies on PCSK9 levels in CKD patients are observational, lacking direct data on the impact of PCSK9 inhibitors on CKD outcomes. Our study fills this gap and validates the conclusions of current reviews, finding no significant correlation between PCSK9 levels and estimated GFR and CKD. Further research is still needed to confirm and elucidate the clinical significance of these observational findings (58).

In MR analysis with eGFR and BUN as outcomes, it was surprising to find that the impact of lipid-lowering drug targets on eGFR was negligible, suggesting that lipid-lowering drugs do not directly affect CKD progression by influencing eGFR levels. The latest meta-analysis of statins and CKD included 33 RCT studies, which similarly found no significant difference in eGFR and serum creatinine levels between the statin group and the control group (18). CKD patients undergoing statin therapy may improve kidney function by reducing urinary albumin and protein excretion or increasing creatinine clearance. The mechanistic insights provided by our mediation analysis further highlight the role of immune regulation in the context of CKD. CD14+ CD16+ monocytes are typically in an activated functional state in CKD patients, contributing to renal inflammation and immune-mediated damage (59). HLA DR, as an important antigen-presenting molecule, reflects the activation level of immune cells (60). In CKD, elevated expression of HLA DR is commonly associated with an overactive immune response and tissue damage. We observed that the protective effect of ANGPTL3 inhibitors may partially mediate through alterations in immune cell phenotypes, particularly the expression of HLA DR on CD14+ CD16+ monocytes. The mediated proportion of this effect was 4.69%, with a mediated effect size of -0.00899, underscoring a potential new pathway by which ANGPTL3 inhibition may confer kidney protection. However, the mediation effect was modest, and the results did not demonstrate significant correlation, warranting cautious interpretation of these findings.

Our study is subject to certain inevitable limitations. Firstly, MR analysis primarily evaluates causal relationships between exposures and outcomes. While it is adept at discerning the direction of these associations, it falls short in quantifying their magnitude and cannot substitute for clinical trials in practical settings. Secondly, drug-targeted MR analysis may not accurately capture the effects of short-term administration or different routes of administration. Lastly, due to limited GWAS data resources, our MR analysis was conducted exclusively on a European population, which may limit the generalizability of our findings to other ethnicities.

## 5 Conclusion

In conclusion, our drug-targeted MR analysis demonstrated that ANGPTL3 inhibitors significantly reduce the risk of CKD, while LDLR activators are significantly associated with an increased risk of CKD. Furthermore, the study found that lipid-lowering drugs do not significantly impact eGFR and BUN levels. These findings suggest that ANGPTL3 and LDLR are promising candidate drug targets for CKD treatment. However, further validation through basic and clinical research is necessary.

## Data Availability

The datasets presented in this study can be found in online repositories. The names of the repository/repositories and accession number(s) can be found in the article/**Supplementary Material**.
