# Supplementary figures and images for: Genetic association analysis of lipid-lowering drug target genes in chronic kidney disease
Source: Front Endocrinol (Lausanne). 2025 Jan 14;15:1434145. doi: 10.3389/fendo.2024.1434145 (PMC11772207; doi:10.3389/fendo.2024.1434145)

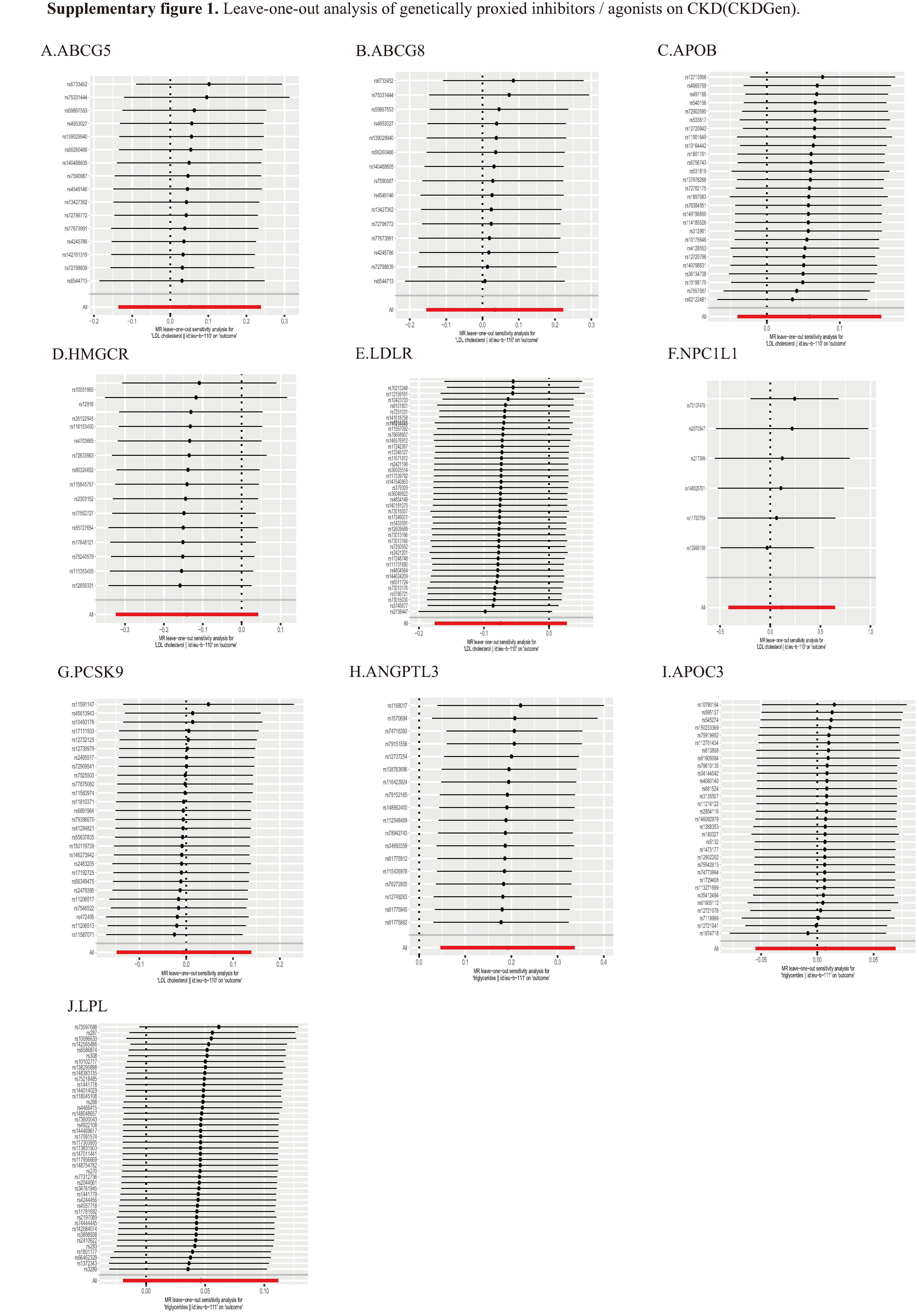

Supplement: Supplementary file 1 [file Image1.tif]

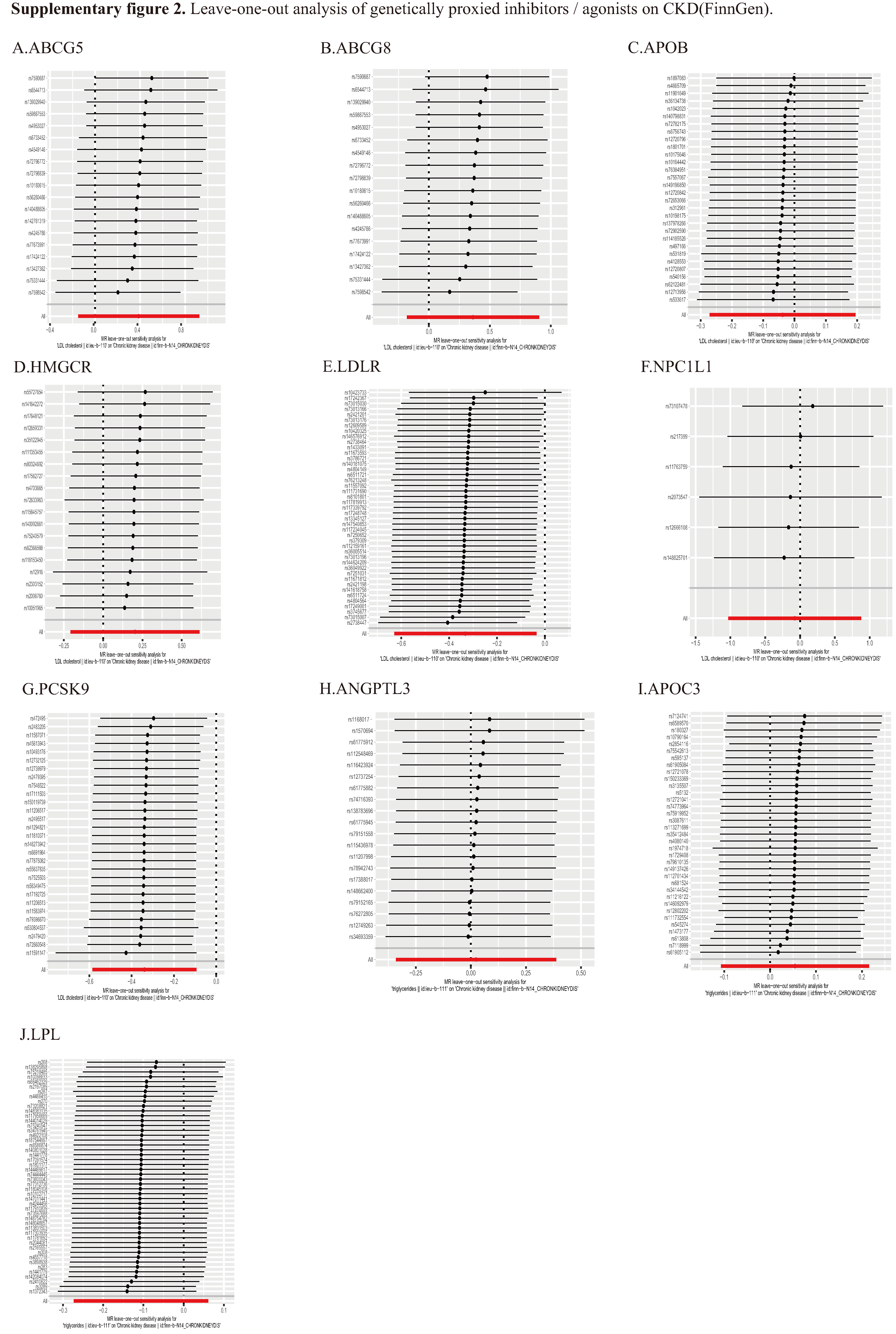

Supplement: Supplementary file 2 [file Image2.tif]

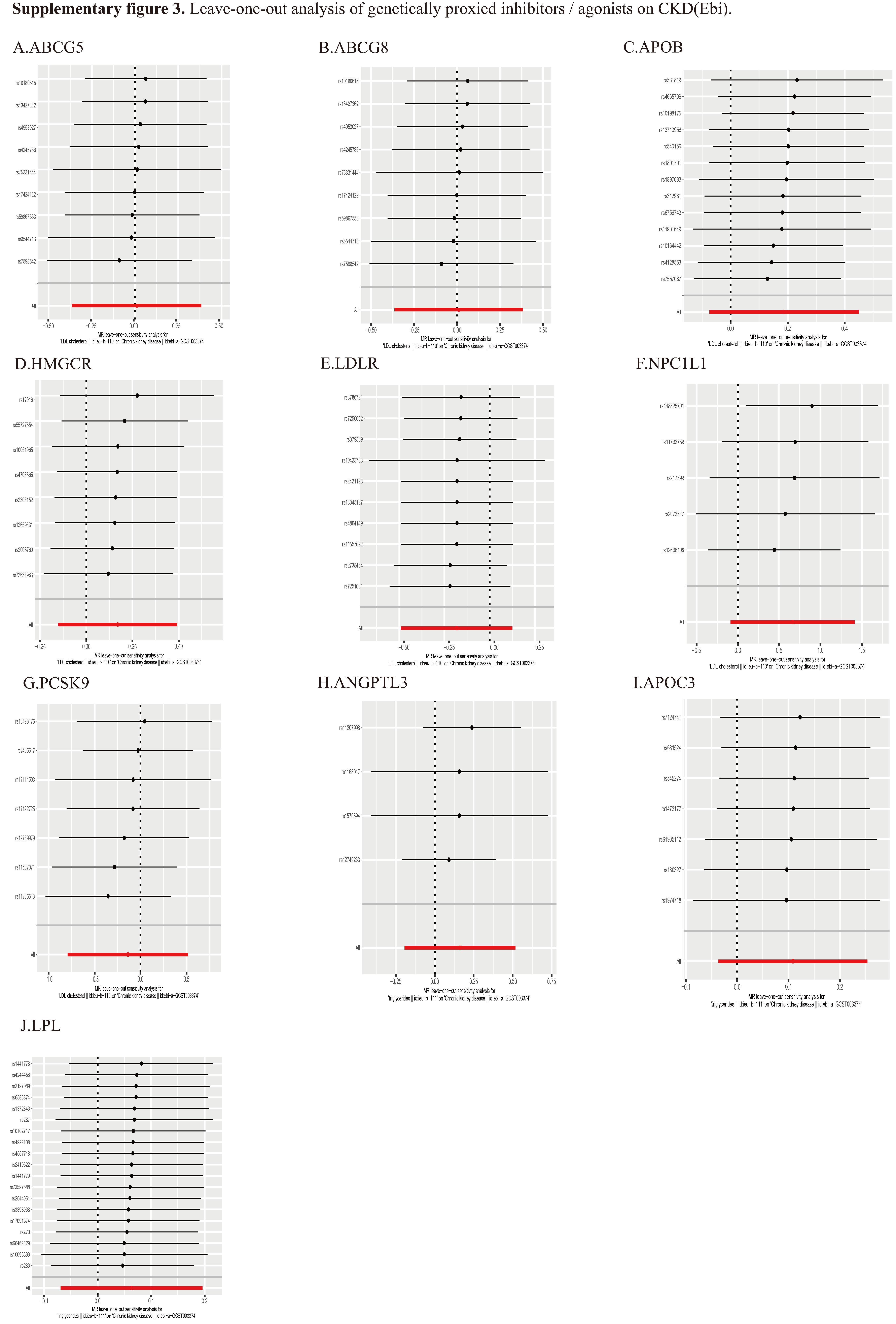

Supplement: Supplementary file 3 [file Image3.tif]

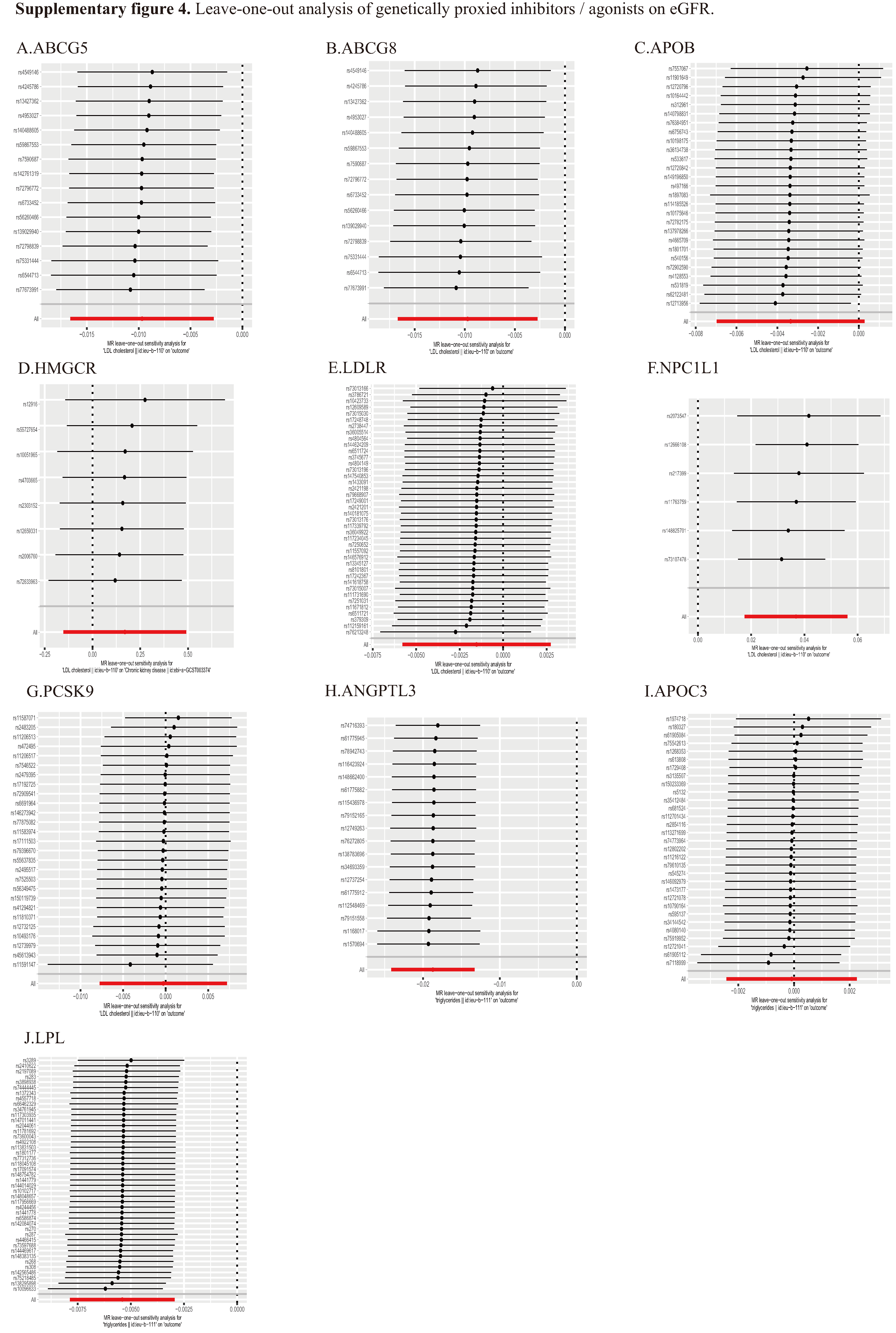

Supplement: Supplementary file 4 [file Image4.tif]

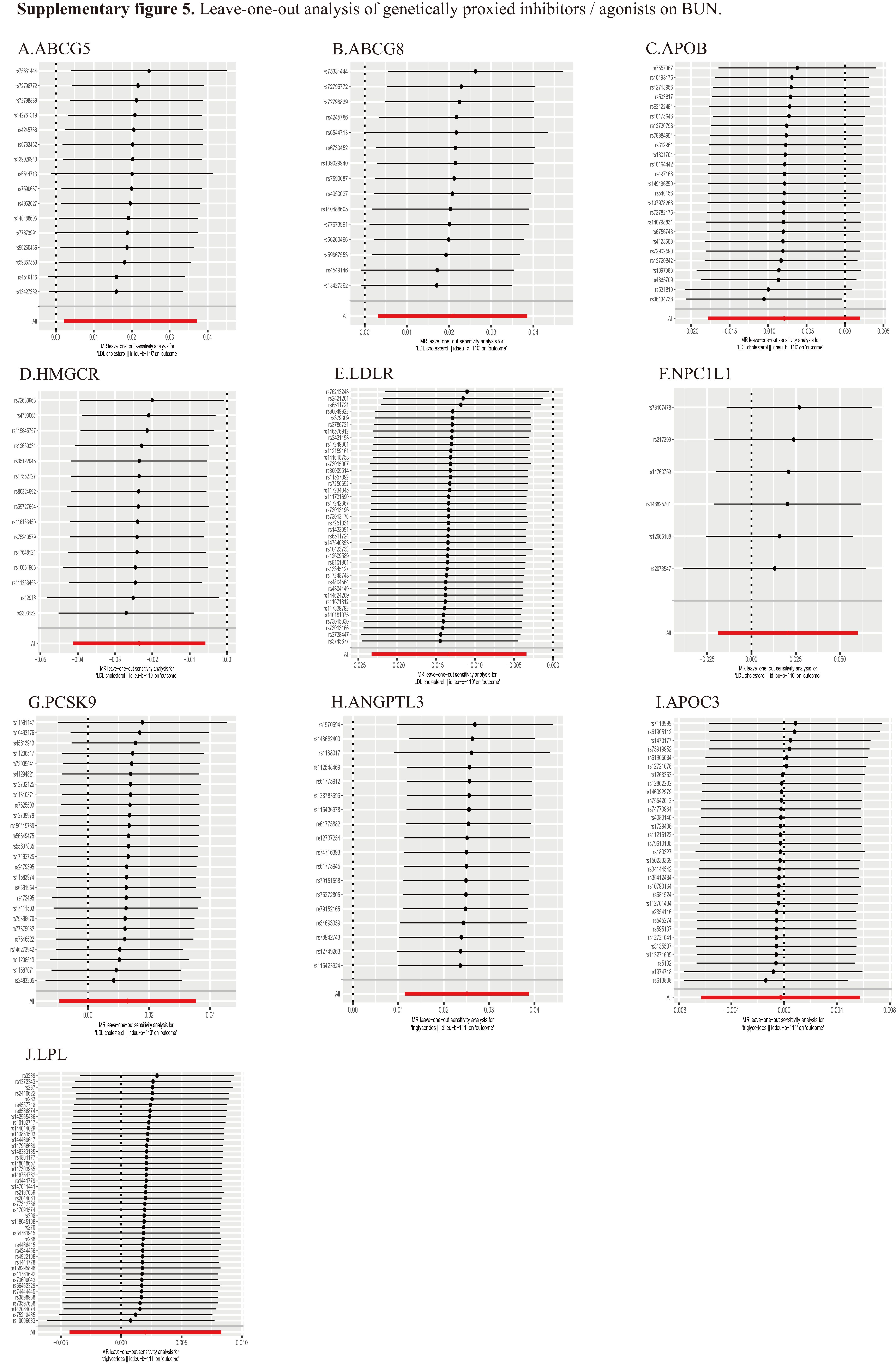

Supplement: Supplementary file 5 [file Image5.tif]

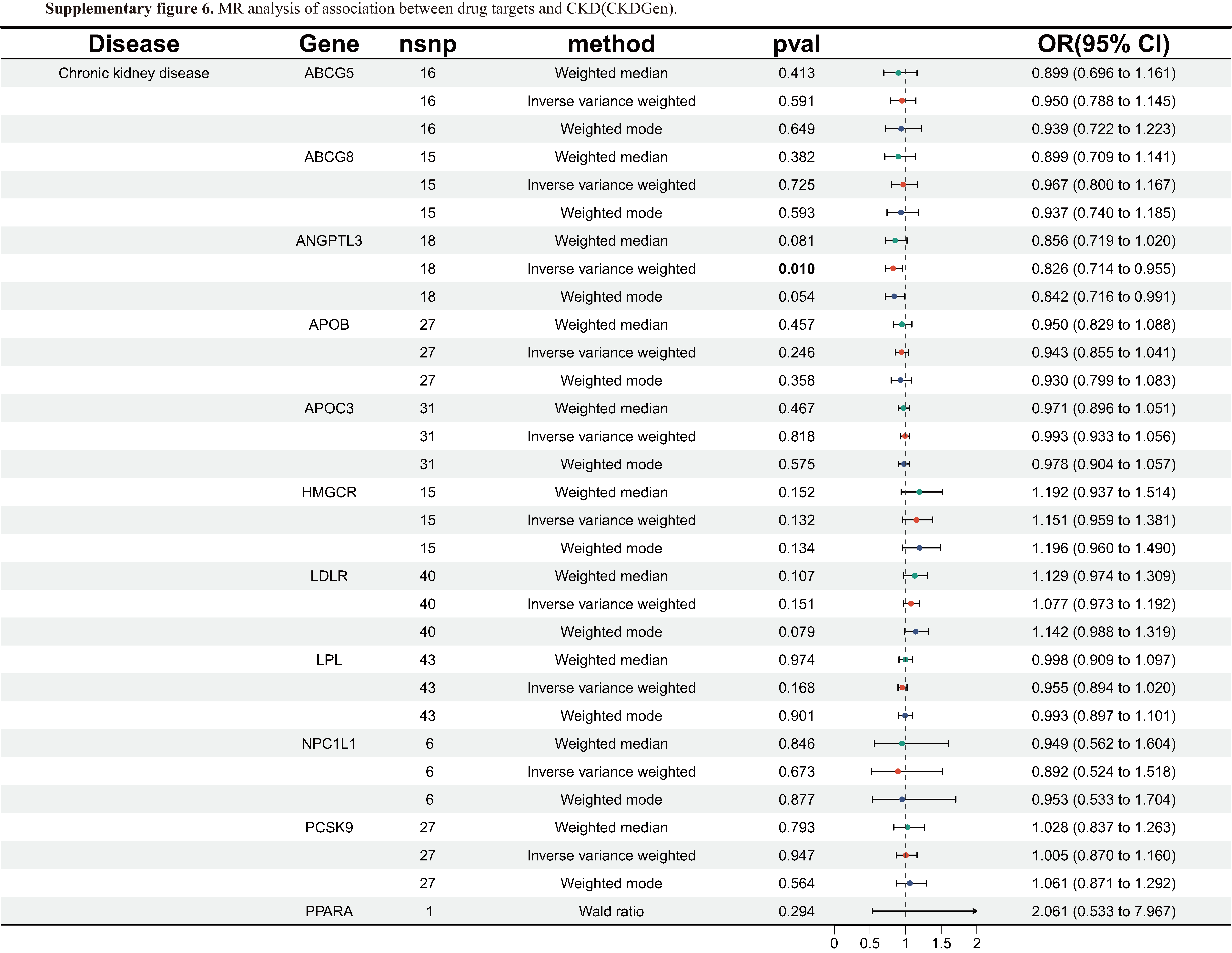

Supplement: Supplementary file 6 [file Image6.tif]

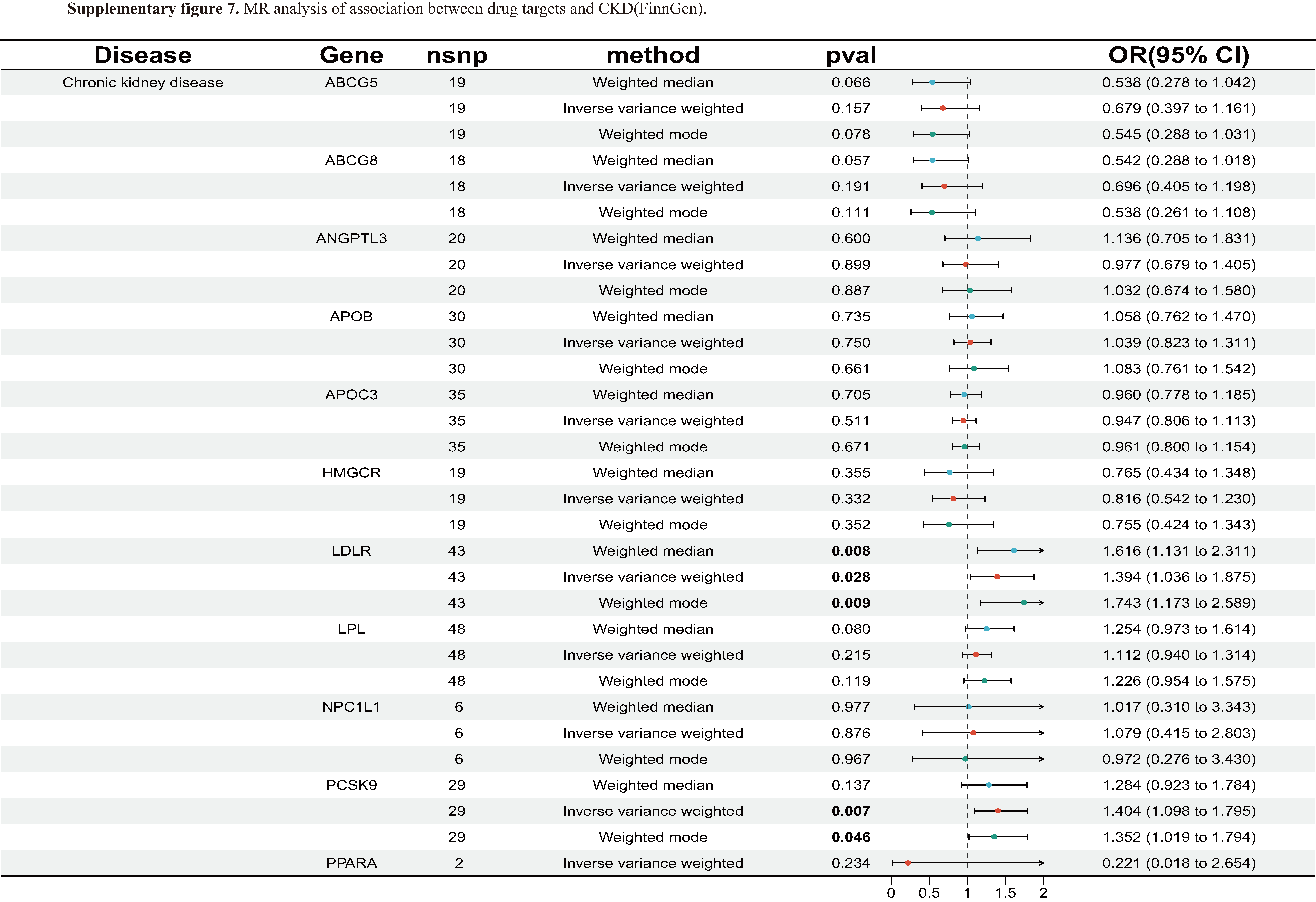

Supplement: Supplementary file 7 [file Image7.tif]

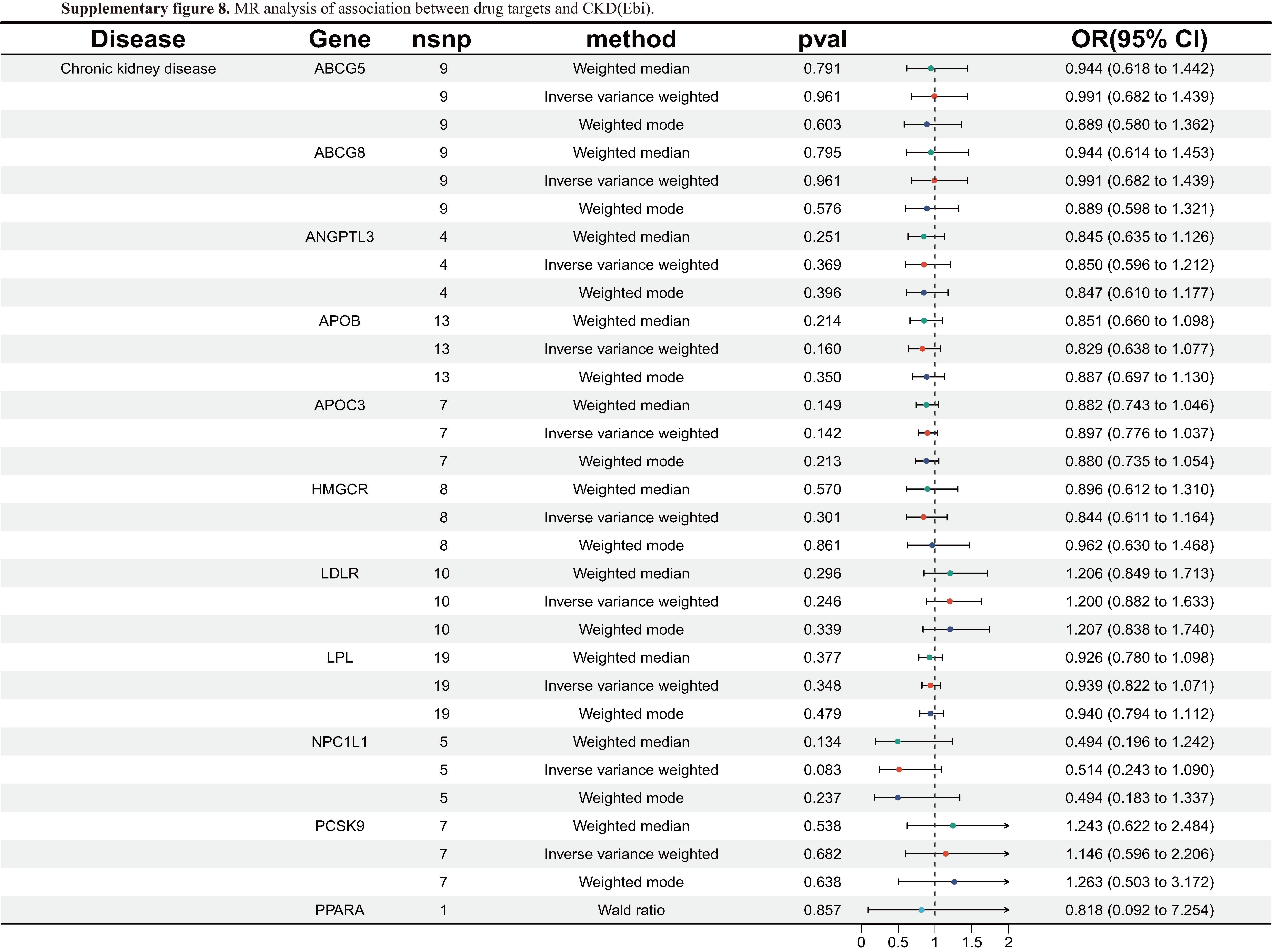

Supplement: Supplementary file 8 [file Image8.tif]
